# Molecular Analysis of BRCA1 in Human Breast Cancer Cells Under Oxidative Stress

**DOI:** 10.1038/srep43435

**Published:** 2017-03-06

**Authors:** Brian L. Gilmore, Yanping Liang, Carly E. Winton, Kaya Patel, Vasilea Karageorge, A. Cameron Varano, William Dearnaley, Zhi Sheng, Deborah F. Kelly

**Affiliations:** 1 Virginia Tech Carilion Research Institute, Virginia Tech, Roanoke, VA, USA; 2 School of Biomedical Engineering and Science, Virginia Tech, Blacksburg, VA, USA; 3 Translational Biology, Medicine, and Health Graduate Program, Virginia Tech, Blacksburg VA, USA; 4 Virginia Tech Carilion School of Medicine, Virginia Tech, Roanoke, VA, USA; 5 Department of Biological Sciences, Virginia Tech, Blacksburg, VA, USA

## Abstract

The precise manner in which physical changes to the breast cancer susceptibility protein (BRCA1) affect its role in DNA repair events remain unclear. Indeed, cancer cells harboring mutations in BRCA1 suffer from genomic instability and increased DNA lesions. Here, we used a combination of molecular imaging and biochemical tools to study the properties of the BRCA1 in human cancer cells. Our results reveal new information for the manner in which full-length BRCA1 engages its binding partner, the BRCA1-associated Ring Domain protein (BARD1) under oxidative stress conditions. We also show how physical differences between wild type and mutated BRCA1^5382insC^ impact the cell’s response to oxidative damage. Overall, we demonstrate how clinically relevant changes to BRCA1 affect its structure-function relationship in hereditary breast cancer.

Germline mutations in the breast cancer susceptibility gene (*BRCA1*) are heavily linked to familial breast and ovarian cancers^1^,^2^,^3^,^4^. Women who inherit these mutations are ~60% more likely to develop the disease^5^,^6^. During normal female development, critical windows of vulnerability correlate with the early onset of breast cancer^7^. These events coincide with a buildup of DNA lesions in mammary tissue as reactive oxygen species are formed from the metabolic processing of estrogen^8^. Damaged DNA, if left unrepaired, can perpetuate errors in the genome^9^. Reduced expression levels of the BRCA1 protein or *BRCA1* mutations, compounded with insufficient lesion repair, provide a tipping point toward cancer induction^10^. At the molecular level, the intricate details of these mechanisms are poorly understood.

In the nucleus, BRCA1 associates with its binding partner the BRCA1-associated Ring Domain protein (BARD1) to help coordinate the repair of DNA modifications. The BRCA1-BARD1 heterodimer performs these operations by interacting with other repair proteins, such as BRCA2, at damaged sites on DNA. In this context, BRCA1 acts as a tumor suppressor to ensure fidelity in the genome^11^,^12^. Inherited mutations in BRCA1 can cause functional deficiencies in the protein that affect BRCA1’s role in tumor suppression^13^,^14^. As decades of scientific research demonstrate BRCA1’s multifaceted role in DNA repair, information about the physical properties of BRCA1 are just coming to light.

While the molecular architecture of the entire BRCA1 protein has not been determined, structural information is available for the BRCA1 N-terminal RING domain^15^ and the C-terminal (BRCT) region^16^, where many clinical mutations reside. Equally important, the manner in which BRCA1 changes in response to cellular stress or inherited mutations remains unclear. Improving our knowledge of BRCA1’s structure-function relationship can provide new insights for therapeutic discovery. Here, we focused on defining differences between wild type and mutated BRCA1 under oxidative stress conditions that contribute to genomic instability. Our results reveal how modifications to BRCA1 influence nuclear events that can weaken DNA repair response.

## Results

### How does oxidative stress effect wild type BRCA1 in breast cancer cells?

**Figure 1:**
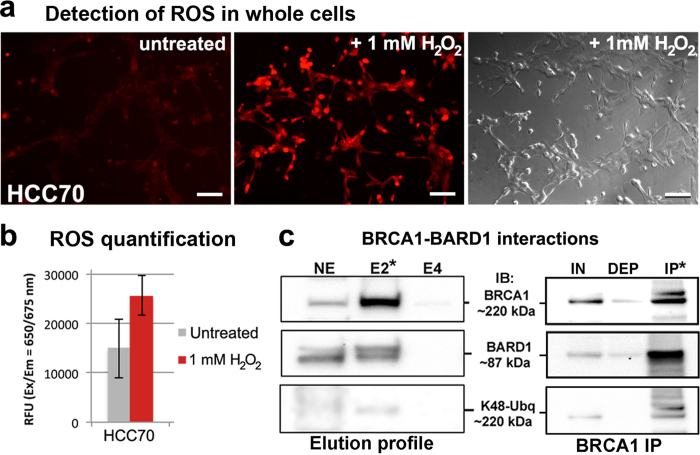
Establishing a breast cancer model system to measure reactive oxygen species (ROS) and wild type BRCA1-BARD1 interactions. (**a**) Upon treating cells with 1 mM H_2_O_2_ for 40 minutes, ROS levels (red) were detected in whole cells using fluorescent microscopy. Scale bar is 100 μm. (**b**) Fluorescence readings indicated less ROS accumulation in untreated than treated cells. Results show mean ± standard deviation. (**c**) Western blot analysis shows wild type BRCA1 (~220 kDa) eluted from Ni-NTA agarose beads in the same fraction (E2) as BARD1 (~87 kDa). K48-linked ubiquitin moieties (K48-Ubq) in the eluted material migrated at ~220 kDa. Co-IP experiments show interactions between BRCA1 and BARD1 in the eluate, and that K48-linked ubiquitin moieties associated with BRCA1. Please see Supplementary Figs 1 and 2 for additional western blots. Nuclear extract (NE); eluted material (E); input material (IN); unbound/depleted material (DEP); immunoprecipitated proteins (IP*).

To investigate the manner in which BRCA1 responds to oxidative stress in breast cancer cells, we employed a combination of molecular imaging and biochemical tools. We first used primary ductal carcinoma cells (HCC70 line^17^; ATCC) known to express wild type BRCA1 and measured their susceptibility to reactive oxygen species (ROS). For these experiments, cells were incubated with culture media containing 1 mM hydrogen peroxide (H_2_O_2_) for up to 1 hour. ROS detection was determined by fluorescent measurements in whole cells using the Cellular Reactive Oxygen Species Detection kit (Deep Red Fluorescence; Abcam). Untreated cells showed minimal background signal throughout the experiments. Differences between H_2_O_2_-treated and untreated cells were detected within 30 minutes (Fig. 1a). These differences were quantified using fluorescence spectroscopy in duplicate experiments, each having four replicates. Greater ROS levels were detected in H_2_O_2_-treated cells compared with untreated cells (Fig. 1b).

We then examined the biochemical nature of BRCA1 and its interactions with BARD1 in HCC70 cells. Cells were collected and lysed, and the cytoplasmic and nuclear material of the lysed cells was separated using the NE-PER kit (Thermo Scientific). Nuclear proteins were enriched by incubating with Nickel-Nitrilotriacetic acid (Ni-NTA)-coated agarose beads for 60 minutes at 4 °C. BRCA1-BARD1 naturally bound to the beads according to western blot analysis. The Ni-NTA elution profile indicated an enrichment of BRCA1 (~220 kDa) and BARD1 (~87 kDa) in fraction E2 along with low levels of K48-linked ubiquitin migrating at ~220 kDa (Fig. 1c; Supplementary Fig. 1). To detect biochemical interactions between these components, we performed co-immunoprecipitation (co-IP) experiments using magnetic beads decorated with IgG antibodies against BRCA1. Western blot detection on the IP material identified interactions between BRCA1 and BARD1 (Fig. 1c; Supplementary Fig. 2). Also, K48-linked ubiquitin moieties co-migrated with BRCA1. The low quantities of K48-linked ubiquitin were a potential indicator of BRCA1 degradation during normal cellular processing events.

### Nuclear BRCA1-BARD1 is relatively stable under oxidative conditions

**Figure 2:**
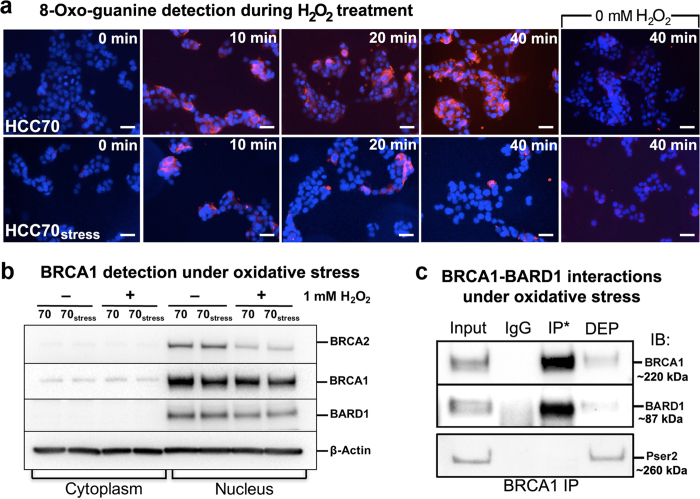
Nuclear wild type BRCA1-BARD1 is relatively stable under oxidative conditions in HCC70 breast cancer cells. **(a)** 8-Oxo-guanine (8-OxoG) accumulation (red) was detected in the nuclei (blue) of HCC70 cells and pre-stressed cells (HCC70_stress_) after treating with 1 mM H_2_O_2_. Fluorescence microscopy detected monoclonal antibodies specific for the 8-OxoG modification. Untreated cells did not show signs of 8-OxoG accumulation during a 40-minute incubation. Scale bar is 100 μm. **(b)** Western blot analysis indicated BRCA2, BRCA1, and BARD1 were primarily found in the nucleus. BRCA2 nuclear levels declined upon H_2_O_2_ treatment while BRCA1 and BARD1 were relatively stable. β-actin served as a loading control. **(c)** Co-IP experiments showed stable interactions between BRCA1 (~220 kDa) and BARD1 (~87 kDa) in the enriched nuclear material of cells experiencing oxidative stress. RNAP II phosphorylated at Pser2 repeats (~260 kDa) served as a negative control. Species-specific IgG control experiments showed low background signal. *denotes immunoprecipitated proteins (IP); unbound/depleted material (DEP); immunoblot (IB). Please see Supplementary Fig. 2 for additional western blots.

Next, we examined the effects of oxidative damage in the cells’ nucleus. Cells were treated with 1 mM H_2_O_2_ and fluorescence microscopy was used to detect antibodies against the oxidated DNA base, 8-Oxo-guanine (8-OxoG) (Santa Cruz Biotechnology). Within 40 minutes of H_2_O_2_ treatment, the signal for 8-OxoG (red fluorescence) increased in and around the nucleus (blue fluorescence) (Fig. 2a, top panels). We also examined 8-OxoG formation in cells that experienced mild thermal stress (HCC70_stress_) prior to H_2_O_2_ treatment. As mild thermal treatment can prime cells to better manage stressful conditions^18^,^19^, HCC70_stress_ cells provided a comparison for oxidative resistance.

Following a 10-minute incubation with H_2_O_2_, 8-OxoG levels increased in all cells. After 20 minutes of treatment, 8-OxoG levels increased in HCC70 cells and this progression continued for up to 40 minutes. Following 60 minutes of treatment, cellular viability limited measurements. In parallel, the HCC70_stress_ cells showed a decrease in 8-OxoG after 20 minutes of treatment and this trend persisted up to 40-minutes (Fig. 2a, bottom panels). Untreated cells showed no signal for 8-OxoG within a 40-minute incubation. Overall, the HCC70 cells were more susceptible to oxidative damage while the pre-stressed HCC70_stress_ cells were more resistant to H_2_O_2_ treatment.

For HCC70_stress_ cells and control cells, BRCA1 and BARD1 were primarily found in the nuclear fractions and in similar quantities. Upon treatment with 1 mM H_2_O_2_, all cells showed a modest reduction in BRCA1 (~18%) and BARD1 (~27%) levels according to densitometry measurements. Unexpectedly, we detected a notable decrease in BRCA2 (~66%), a known DNA damage response protein (Fig. 2b).

We further evaluated BRCA1’s ability to interact with BARD1 under oxidative stress conditions. Co-IP results showed that BRCA1 associated with BARD1 in high quantities (Fig. 2c; Supplementary Fig. 2). Control experiments using species-specific IgG antibodies or probing for phosphorylated (pSer2) RNA polymerase II (RNAP II), showed little to no protein association (Fig. 2c, bottom row; Supplementary Fig. 2). Experiments performed on control cells showed approximately the same level of interaction as the stressed cells (similar to Fig. 1c). These results indicated that wild type BRCA1 was relatively stable under oxidative conditions in the nucleus, and that its association with BARD1 was strongly maintained.

### The BRCA1^5382insC^ mutation influences cellular susceptibility to oxidative damage

**Figure 3:**
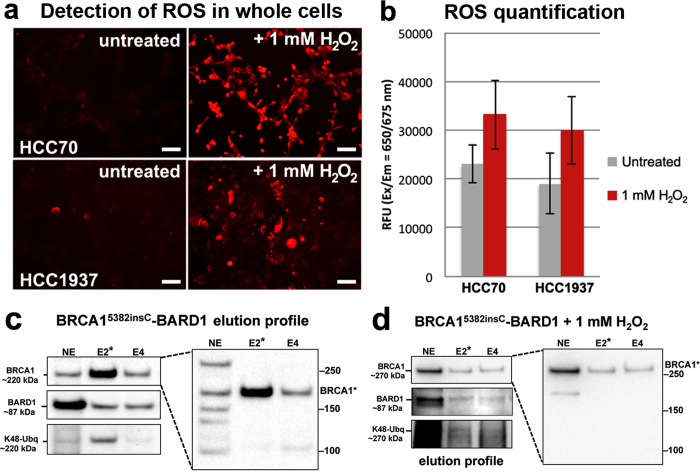
The BRCA1^5382insC^ mutation influences cellular susceptibility to oxidative damage and changes in protein mobility. **(a)** ROS accumulation (red fluorescence) was detected and **(b)** quantified for HCC1937 cells and HCC70 control cells. Results show mean ± standard deviation. Scale bar is 100 μm. **(c)** In untreated HCC1937 cells, western blots show BRCA1^5382insC^ (~220 kDa) eluted from Ni-NTA agarose beads in the same fractions (E2–E4) as BARD1 (~87 kDa). K48-linked ubiquitin moieties (K48-Ubq) in the eluted material migrated at ~220 kDa. **(d)** In H_2_O_2_-treated HCC1937 cells, western blot analysis reveals BRCA1^5382insC^ (~270 kDa) eluted from Ni-NTA agarose beads in the same fractions (E2–E4) as BARD1 (~87 kDa). K48-linked ubiquitin moieties (K48-Ubq) were detected at ~270 kDa, having the same shift in mobility as BRCA1^5382insC^. Please see Supplementary Fig. 3 for additional western blots. Nuclear extract (NE); Eluted material (E).

Next, we examined cells that produced the prevalent BRCA1^5382insC^ cancer-related mutation. We hypothesized that mutated BRCA1^5382insC^ produced in the HCC1937 breast cancer line^20^ (ATCC) may affect the physical properties and functional capacity of the protein. We assessed global ROS values in HCC1937 cells using fluorescence imaging experiments performed on whole cells. We detected similarly low starting amounts of ROS in HCC1937 and HCC70 cells expressing wild type BRCA1. After treating cells with 1 mM H_2_O_2_ for 40 minutes, ROS levels increased in treated cells compared to control cells evaluated across duplicate experiments, each having four replicates (Fig. 3a). Quantifying these results, we found HCC1937 cells were susceptible to oxidative damage and ROS formation similar to HCC70 cells expressing wild type BRCA1 (Fig. 3b).

Next, we examined the biochemical nature of the mutated BRCA1^5382insC^ protein produced in HCC1937 cells. Treated cells were collected and lysed following a 40-minute incubation with H_2_O_2_. Control cells were incubated with media lacking H_2_O_2_ and processed in the same manner. Nuclear proteins were separated and enriched using the same procedure implemented for wild type BRCA1. Western blot analysis revealed that BRCA1^5382insC^, which is truncated by ~10 kDa, migrated the same as wild type BRCA1 in untreated cells (Fig. 3c). Physical changes or modifications to BRCA1^5382insC^ may account for this slower than expected migration. K48-linked ubiquitin moieties co-migrated with BRCA1^5382insC^ at ~220 kDa (Fig. 3c; Supplementary Fig. 3a,b).

Western blot analysis of the H_2_O_2_-treated cells identified BRCA1^5382insC^ migrated at a much slower rate (~270 kDa) than wild type BRCA1 (~220 kDa) (Fig. 3d; Supplementary Fig. 3a,b). This shift in mobility also corresponded with a shift in K48-linked ubiquitin moieties (~270 kDa). In both treated and untreated cells intact BARD1 migrated at ~87 kDa although its detection was more complex in the treated cells (Supplementary Fig. 3c). Taken together, these results suggested that oxidative damage can enhance K48-linked ubiquitin attachments to mutated BRCA1^5382insC^.

### How does the BRCA1^5382insC^ mutation affect protein stability in the nucleus?

**Figure 4:**
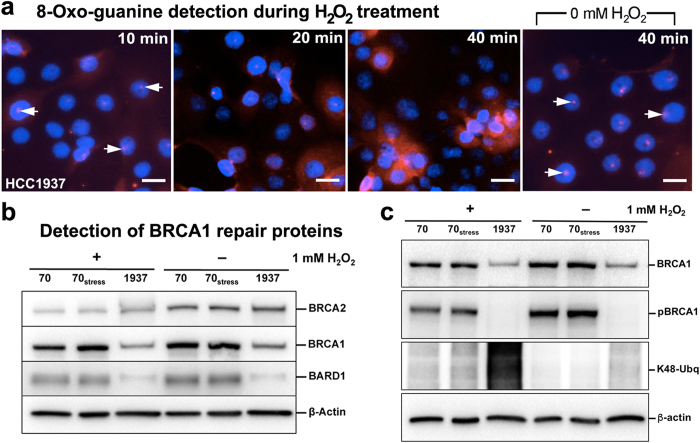
Breast cancer cells expressing BRCA1^5382insC^ show deficiencies in DNA damage repair and changes in nuclear protein properties. (**a**) Foci of 8-OxoG (red, white arrows) were detected in the nucleus (blue) of HCC1937 within 10 minutes of H_2_O_2_ treatment. 8-OxoG lesions accumulated over 40 minutes. Untreated cells showed background levels of 8-OxoG nuclear foci that persisted for 40 minutes. Scale bar is 50 μm. (**b**) Western blot analysis showed more stable BRCA2 levels in HCC1937 cells upon H_2_O_2_ treatment, and lower levels of BRCA1 and BARD1. Wild type BRCA1 and BARD1 in HCC70 and HCC70_stress_ cells showed only minor changes upon H_2_O_2_ treatment. β-actin served as a loading control. (**c**) The nuclear material of H_2_O_2_-treated and untreated cells including HCC70, HCC70_stress,_ and HCC1937 lines was assessed by western blot analysis. Polyclonal antibodies against the BRCT domain (top panel) indicated the presence of BRCA1 in each fraction. Phosphorylated BRCA1 (pBRCA1) was detected in HCC70 and HCC70_stress_ cells with and without H_2_O_2_ treatment using monoclonal antibodies against phosphorylated residue S1524. pBRCA1 was not detected in HCC1937 cells. K48-linked ubiquitin moieties were elevated in all cells upon H_2_O_2_ treatment, particularly in HCC1937 cells. β-actin served as a loading control. Please see Supplementary Fig. 4 for additional western blots.

To gain insight on the functional nature of BRCA1^5382insC^ during DNA damage response, we treated HCC1937 cells with 1 mM H_2_O_2_ for up to 40 minutes and used fluorescence microscopy to detect 8-OxoG accumulation. Treated HCC1937 cells showed the same trend as treated HCC70 cells. We detected 8-OxoG in and around the nucleus within 20 minutes and levels continued to accumulate for up to 40 minutes (Fig. 4a). Untreated cells showed evidence of 8-OxoG at the end of the 40-minute incubation period and some signal was also detected at the start of the experiment (Fig. 4a, white arrows). These results indicated that cells expressing BRCA1^5382insC^ were not well-equipped to manage oxidative conditions in the nucleus.

We then investigated how BRCA1^5382insC^ and its binding partners were biochemically effected by oxidative effects. We evaluated nuclear levels of BRCA1, BARD1, and BRCA2 in different cell lines using western blot analysis and densitometry measurements performed on three replicate experiments (Fig. 4b). Treated HCC1937 cells exhibited more stable levels of BRCA2 than the other cells. By comparison, BRCA1 levels were reduced in treated HCC1937 cells by ~50% relative to the other cells. BARD1 levels in the HCC1937 cells were lowered by ~80%. Consistent with western blot results in Fig. 3, BRCA1^5382InsC^ in H_2_O_2_-treated cells also migrated slower than wild type BRCA1. We posited these changes were due to modifications to BRCA1^5382InsC^ that occurred under oxidative conditions. Specifically, we found an increase in K48-linked ubiquitination in the nuclear extract of treated HCC1937 cells. The enhanced K48-ubiquitin signal corresponded with a mobility shift in BRCA1^5382InsC^ in treated cells (Figs 3d and 4c). An accumulation of K48-linked ubiquitin adducts on BRCA1^5382insC^ can cause the mutated protein to be degraded at higher levels. This mechanism would account for the reduced amounts of BRCA1 found in the treated HCC1937 cells. While other forms of ubiquitination or phosphorylation were also feasible they were not detected (Fig. 4c; Supplementary Fig. 4).

### The BRCA1^5382insC^ structure is modified under oxidative conditions

**Figure 5:**
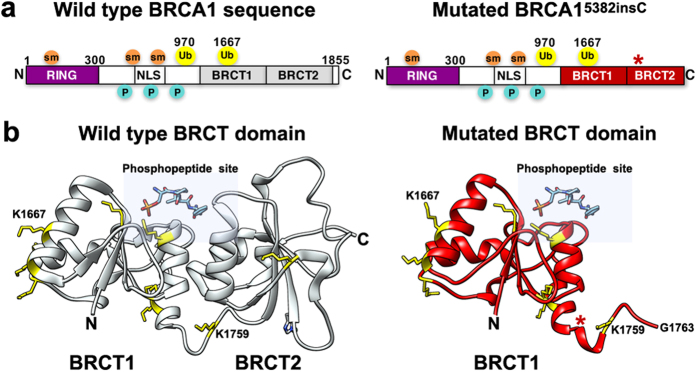
Post-translational modifications to the BRCA1 structure and potential ubiquitination sites in the C-terminal region. **(a)** A comparison of the wild type and mutated BRCA1 primary sequence with some modifications noted for sumoylation (sm; orange), phosphorylation (P; cyan), and ubiquitination (Ub; yellow). The N-terminal RING domain (magenta) is followed by an unstructured central region containing nuclear localization sequences (NLS) and the structured C-terminal region comprised of BRCT1 and BRCT2 (gray for wild type, red for mutant). Lysine residues 970 and 1667 were identified as ubiquitination sites in proteomic analysis^21^,^22^. **(b)** Crystal structure (gray) for the wild type BRCT domain bound to it cognate phosphopeptide substrate (pSPTF) (pdb code, 3K0H^23^). All lysine resides are colored yellow with stick rendering and labels are given for K1667 and K1759. For comparison, a homology model (red) for the mutated BRCT domain of BRCA1^5382insC^ is shown. The BRCT2 domain is unstructured and truncated shortly after residue K1759, which is more accessible in the truncated protein. The phosphopeptide binding site is highlighted with a blue rectangle. Red star indicates the mutation site in BRCA1^5382insC^.

To understand the physical consequences of oxidative damage to BRCA1, we examined documented sites for post-translational modifications using the online bioinformatics tool, PhosphoSitePlus^21^ (http://www.phosphosite.org/). Identified modifications to human BRCA1 include phosphorylation, ubiquitination, sumoylation, and acetylation (Fig. 5a). Although there are many lysine residues dispersed throughout the BRCA1 sequence, specific lysines have been documented through mass spectrometry analysis as ubiquitination sites^21^,^22^. These residues include K970 and K1667. There is currently no structural information available for the region of BRCA1 that encompasses K970. Residue K1667 is located in the BRCA1 C-terminal region (pdb code, 3K0H)^23^ known as the BRCT1 domain (Fig. 5b). This location is ~200 residues prior to the end of the BRCA1 sequence and is close to the phosphopeptide binding site.

The mutated BRCA1^5382insC^ protein sequence (Fig. 5a) can be translated and modeled up to residue G1763^24^,^25^. After this glycine residue, mutated BRCA1 shows no secondary structure and is truncated in the BRCT2 domain (Fig. 5b). The truncated protein is also thought to be misfolded to some extent, which can trigger K48-linked ubiquitination and proteasomal degradation^26^. The proximity of K1667 to the mutation site (Fig. 5b, red star) makes it a prime candidate for ubiquitination in response to errors in protein folding. Similarly, residue K1759 is located in the connecting loop between BRCT1 and BRCT2, and it is more accessible in the mutated BRCA1^5382insC^ structure. This gain in accessibility also makes K1759 a strong candidate for ubiquitination in mutated BRCA1^5382insC^ (Fig. 5b).

We biochemically tested for other modifications to BRCA1^5382insC^ including phosphorylation and sumoylation. No phosphorylation signal was detected on western blot analysis when probing for these modifications, especially S1524 (Fig. 4c). This finding is consistent with the work of Okada and Ouchi^27^. Sumoylation was also not noted in our analysis, which is not surprising considering the transient nature of these modifications and the fact that H_2_O_2_ can prevent these attachments from occurring^28^.

## Discussion

In summary, we present biochemical and molecular imaging information to explain differences in wild type and mutated BRCA1 under oxidative conditions. We found notable changes in the physical properties of mutated BRCA1 related to ubiquitination patterns and the molecular architecture of the BRCT region. Under oxidative conditions, mutated BRCA1^5382insC^ exhibited a slower than expected mobility according to western blot analysis. A shift in BRCA1^5382insC^ migration corresponded with changes in K48-linked ubiquitin migration. These observations support the idea that modifications to BRCA1^5382insC^ were enhanced in cells experiencing oxidative damage.

To better understand the manner in which modifications to BRCA1 can affect its function, we assessed ROS accumulation in whole cells and in the nucleus. Cells expressing wild type or mutated BRCA1 were susceptible to oxidative stress. However, cells producing BRCA1^5382insC^ showed a major decline in proteins levels and inherent amounts of oxidated DNA bases. These results indicate long-term genomic instability and insufficient lesion repair in cells harboring the BRCA1^5382insC^ mutation. Our findings are consistent with other reports for BRCA1^5382insC^ and may account for its documented deficiencies in protein-protein interactions^29^,^30^. Cells expressing wild type BRCA1 showed relatively stable protein levels and lacked inherent oxidated DNA bases. These observations underscore the functional importance of BRCA1-BARD1 in responding to oxidative conditions in the nucleus.

To place our results in the context of other studies, we looked to researchers who have tested the ubiquitin ligase activity of BRCA1. Studies performed on cells expressing different BRCA1 constructs demonstrated the importance of the RING domain in mediating ubiquitin transfer events. Specifically, the I26A mutation located in the RING domain tends to limit BRCA1’s association with E2 binding partners and reduce its ubiquitination capacity^29^. Other studies demonstrated that the I26A mutant expressed in HCC1937 cells, although deficient in ligase activity, exhibited the same physical properties and mobility as wild type BRCA1 in rescue experiments^30^. Still, others have shown that BRCA1^5382insC^ has natural deficiencies in ubiquitin ligase activity due to inadequacies in the mutated BRCT domain^31^,^32^. There is no compelling evidence in the literature to indicate BRCA1 transfers K48-type ubiquitin moieties to protein substrates, rather K6-type ubiquitin moieties are typically transferred^33^,^34^,^35^,^36^,^37^. Therefore, the enhanced modifications to BRCA1^5382insC^ formed in HCC1937 cells under oxidative conditions are unique and not likely due to ubiquitin intermediates associated with its E3-ligase function.

Another explanation for the changes to BRCA1^5382insC^ may be related to auto-ubiquitination. Extensive work by other researchers demonstrates enhanced BRCA1 activity through auto-ubiquitination mechanisms also involving K6-type ubiquitin linkages^33^,^35^,^36^,^38^. According to our oxidative damage response assays, reduced BRCA1 activity was observed as protein levels declined. In the case of the BRCA1^5382insC^ mutation, protein levels were further diminished upon modification. Therefore, our results do not support the idea of auto-ubiquitination to mutated BRCA1^5382insC^. Rather, they point to differences in protein stability levels of BRCA1^5382insC^ in response to ROS accumulation. Our working hypothesis is that protein misfolding in BRCA1^5382insC^ triggers an increase in K48-linked ubiquitination, possibly within the BRCT domain, and that this effect is enhanced by oxidative stress.

Collectively, we demonstrate that protein levels, genetic mutations, and post-translational modifications – particularly ubiquitination – can affect BRCA1’s functional state in breast cancer cells. Our present work puts forth the idea of testing mechanistic-based therapies such as deubiquitinating enzymes to restore the physical properties of mutated BRCA1^5382insC^ in cells. Future structural studies on full-length BRCA1 can complement our findings to better inform us of the physical variations in BRCA1 related to clinically harmful mutations.

## Methods

### Authentication and preparation of cells and enriched nuclear fractions

Breast cancer cells (HCC70 and HCC1937 lines) were were purchased from the American Type Culture Collection (ATCC) and independently characterized by ATCC as being primary ductal carcinoma cells and triple negative in nature (i.e., lacking expression of estrogen receptor, progesterone receptor, and Her2). Cells were used within 6 months of resuscitation. Cells were grown in complete growth medium (RPMI-1640; Mediatech) supplemented with 10% fetal bovine serum (ATCC) and 0.5X penicillin-streptomycin (Thermo Fisher) at 37 °C and 5% CO_2_ until confluent. Matched HCC70 cells were stressed by the addition of complete media warmed to 24 °C, then returned to 5% CO_2_ and 37 °C for ~5–7 cycles. Control cells having a similar passage number were cultured with media warmed to 37 °C. For each experiment, we collected ~1,000,000 cells using Trypsin-EDTA (Thermo Fisher) followed by centrifugation (500× *g*, 5 minutes). The resulting cell pellet was washed with PBS followed by re-pelleting. The NE-PER kit (Thermo Scientific) was used to separate cytoplasmic and nuclear fractions. The nuclear fractions were diluted (~1 mg/ml) in 20 mM HEPES buffer (pH 7.2) containing 2 mM MgCl_2_, 2 mM CaCl_2_, 5 mM imidazole and Complete protease inhibitor cocktail (EDTA-free, Roche). BRCA1-BARD1 heterodimers were enriched from the soluble nuclear fraction by incubating with pre-equilibrated Nickel-Nitrilotriacetic acid (Ni-NTA) agarose beads (Qiagen) for 1 hour at 4 °C with rotation. The Ni-NTA beads were washed with 20 mM HEPES buffer (pH 7.2) containing 140 mM NaCl_2_, 5 mM imidazole and proteins were eluted in the same buffer having 150 mM imidazole. Protein concentrations were estimated using the Pierce Bradford assay (Thermo Scientific).

### Fluorescence imaging of 8-OxoG DNA lesions

Cells (~50,000 per chamber) were seeded in an eight-chamber slide and incubated at 37 °C and 5% CO_2_ overnight. To induce oxidative damage in cells, 1 mM hydrogen peroxide (H_2_O_2_) (Sigma) was added to culture media and cells were examined at different time points up to 1-hour post-treatment. Cells were washed with standard PBS solution then fixed with 4% paraformaldehyde (Electron Microscopy Sciences) for 15 minutes. Cells were permeabilized by incubating with PBS solution containing 0.5% Triton X-100 (Sigma) for 10 minutes followed by a 1-hour blocking step with PBS supplemented with 10% normal goat serum (Jackson Immuno Research, #005–000–121) and 0.2% Triton X-100. Control cells were treated with complete culture media lacking H_2_O_2_. Cells were incubated with anti-8-oxoG DNA Lesion (483.15) (Santa Cruz, #sc-130914) at 4 °C overnight. Following a wash step with PBS, antibodies were detected with goat anti-mouse IgM-TR (Santa Cruz, #sc-2983). Nuclei were stained with Hoechst 33342 and cells were imaged using an inverted fluorescence microscope (Zeiss Axio Vert.A1; Carl Zeiss Microscopy).

### ROS detection and quantitation

To detect and quantify levels of ROS accumulation in whole cells, we used the Cellular Reactive Oxygen Species Detection Assay Kit (Deep Red fluorescence; Abcam, #ab186029). Cells (~20,000 per well) were plated in a 96-well black wall/clear bottomed microplate and incubated at 37 °C and 5% CO_2_ overnight. Cells were treated with 1 mM hydrogen peroxide (H_2_O_2_) in standard PBS solution containing 10% Fetal Bovine Serum (FBS) for 40 minutes. In parallel, control cells were treated with the same PBS solution lacking H_2_O_2_ for 40 minutes. Treated and control cells were incubated in ROS Deep Read working solution for 30–60 minutes in the dark at 37 °C and 5% CO_2_. The deep red fluorescence was read using the SpectraMax i3 microplate reader (at excitation 650 nm, emission 675 nm; Molecular Devices). Cells were imaged using an inverted fluorescence microscope.

### Co-IP experiments and immunoblot analysis

For co-immunoprecipitation (IP) experiments, antibodies against BRCA1 (5 μg Santa Cruz Biotechnology (SCBT); sc-642, C-20) or normal rabbit IgG (5 μg Cell Signaling Technology; 2729) were diluted in PBS-T (0.02% Tween-20) before incubating with 0.75 mg Dynabeads Protein G (Thermo Fisher). The mixtures were incubated with rotation for 30 minutes at 4 °C. Antibody-coated beads were washed in 20 mM HEPES buffer (pH 7.2) containing 140 mM NaCl_2_ prior to adding enriched nuclear fractions. Eluates from the Ni-NTA beads were pooled (200 μg per experiment) and supplemented with protease and phosphatase inhibitor cocktails (Thermo Fisher). This material was incubated with the antibody-coated beads overnight at 4 °C with gentle rotation. The beads were then washed followed by elution with NuPAGE LDS sample buffer (Thermo Fisher).

Western blot analysis was performed to identify proteins separated on 3–8% Tris-Acetate NuPAGE mini gels (Thermo Fisher) and transferred onto Immobilon-P membranes (Millipore) in a Mini-PROTEAN Tetra system (Bio-Rad). Blots were blocked with 1% non-fat dry milk (NFDM) in TBS-T (0.05%) for 1 hour with gentle rocking. Primary antibodies were diluted in blocking solution and incubated overnight at 4 °C. The following primary antibodies were used in our studies: BRCA1 (SCBT; sc-642, C-20), pBRCA1 (S1524-specific; Bethyl, A300–001A), BRCA2 (EMD Millipore; OP95), BARD1 (SCBT; sc-11438), RNA Polymerase II (Pser2-specific; Covance MMS-129, H5), ubiquitin (K48-linkage-specific, Abcam, ab140601), and β-Actin (Sigma Aldrich; A5441). Blots were washed three times with TBS-T (0.05%). Goat anti-rabbit, goat anti-mouse, mouse anti-rabbit (light chain-specific, IP), or goat anti-mouse (light chain-specific, IP) secondary antibodies conjugated to horseradish peroxidase (Jackson ImmunoResearch) were incubated for 1 hour followed by additional washing. ECL Prime western blotting reagent (GE Healthcare) or West Femto (Thermo Fisher) was used for detection. A ChemiDoc MP (Bio-Rad) was used for imaging and densitometry.

### Proteomic and homology modeling analysis

To evaluate documented post-translational modifications to the BRCA1 primary sequence, we utilized the PhosphoSitePlus website, which is freely available at http://www.phosphosite.org/. The human isoform of BRCA1 (ACC_ID, P38398) was used in our analysis. Ubiquitination results were reported based on the number of records in which this modification was assigned using proteomic discovery-mode mass spectrometry. The homology model presented here was generated using the online SWISS-MODEL website (http://swissmodel.expasy.org) and was previously reported^24^,^25^.

## Additional Information

**How to cite this article**: Gilmore, B. L. *et al*. Molecular Analysis of BRCA1 in Human Breast Cancer Cells Under Oxidative Stress. *Sci. Rep.* **7**, 43435; doi: 10.1038/srep43435 (2017).

**Publisher's note:** Springer Nature remains neutral with regard to jurisdictional claims in published maps and institutional affiliations.

## Supplementary Material

Supplementary Information
