# Supplementary material for: Molecular Analysis of BRCA1 in Human Breast Cancer Cells Under Oxidative Stress
Source: Sci Rep. 2017 Mar 6;7:43435. doi: 10.1038/srep43435 (PMC5338271; doi:10.1038/srep43435)
Supplement: Supplementary Information [file srep43435-s1.pdf]

Molecular Analysis of BRCA1 in Human Breast Cancer Cells Under Oxidative Stress

Brian L. Gilmore[1], Yanping Liang[1], Carly E. Winton[1,2], Kaya Patel[1], Vasilea Karageorge[1], A. Cameron Varano[1,3], William Dearnaley[1], Zhi Sheng[1,2,4], and Deborah F. Kelly[1,2,4,5*]

[1]Virginia Tech Carilion Research Institute, Virginia Tech, Roanoke, VA

[2]School of Biomedical Engineering and Science, Virginia Tech, Blacksburg, VA

[3]Translational Biology, Medicine, and Health Graduate Program, Virginia Tech, Blacksburg VA

[4]Virginia Tech Carilion School of Medicine, Virginia Tech, Roanoke, VA

[5]Department of Biological Sciences, Virginia Tech, Blacksburg, VA

***Correspondence to**: Deborah F. Kelly, Virginia Tech Carilion Research Institute, 2 Riverside Circle, Roanoke, VA 24016. Phone: 540-526-2031; Fax: 540-985-3373; Email: debkelly@vt.edu

Supplementary information

Supplementary Figures

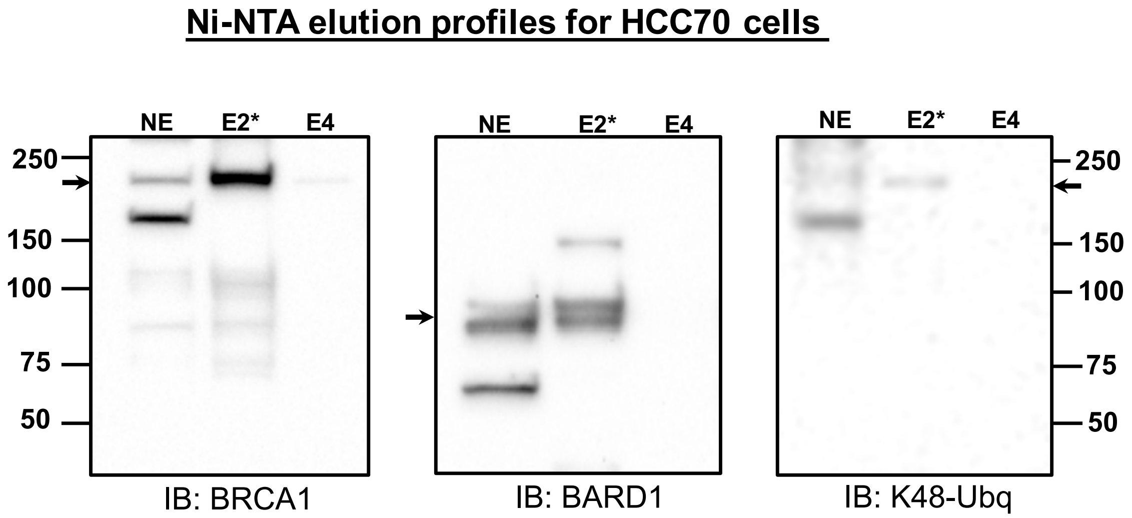

Supplementary Figure 1. Western blot analysis of Ni-NTA elution profiles for HCC70 cells. Western blot analysis indicates wild type BRCA1 (~220 kDa) eluted from Ni-NTA agarose beads in the same fraction (E2) as BARD1 (~87 kDa). Minor quantities of K48-linked ubiquitin moieties (K48-Ubq) were detected in the eluted material that migrated at ~220 kDa. Nuclear extract (NE); eluted material (E).

BRCA1-BARD1 Co-IP results for HCC70 cells

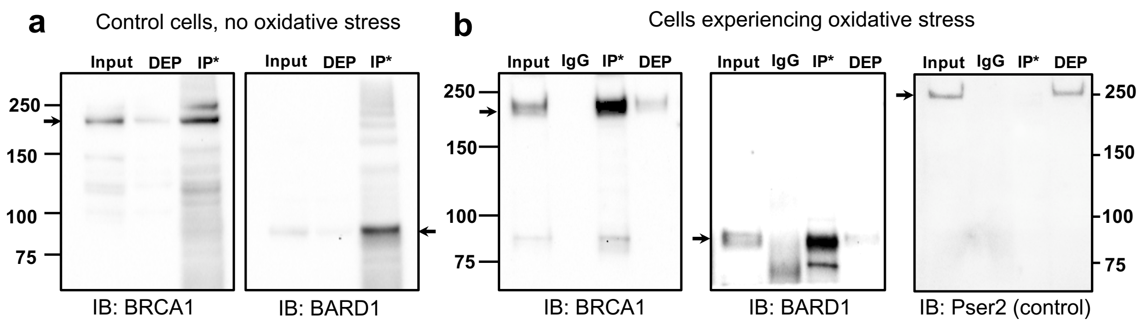

Supplementary Figure 2. BRCA1 Co-IP experiments performed in HCC70 cells. (a) Co-IP experiments showed interactions between BRCA1 (~220 kDa) and BARD1 (~87 kDa) in the Ni-NTA eluate in HCC70 control cells. **(b)** Co-IP experiments showed stable interactions between BRCA1 (~220 kDa) and BARD1 (~87 kDa) in the enriched nuclear material of cells experiencing oxidative stress. RNAP II phosphorylated at Pser2 repeats (~260 kDa) served as a negative control. Species-specific IgG control experiments showed low background signal.*denotes immunoprecipitated proteins (IP); unbound / depleted material (DEP); immunoblot (IB).

Ni-NTA Elution Profiles for HCC1937 cells

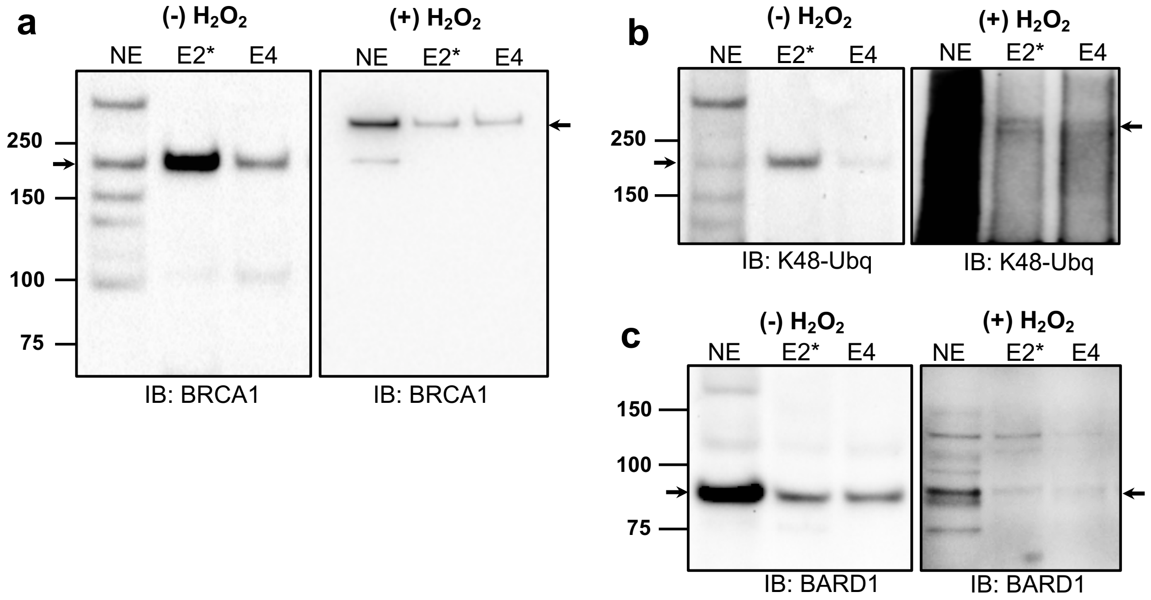

Supplementary Figure 3. Ni-NTA elution profiles for HCC1937 cell in the presence and absence of 1 mM H_2O_2 treatement. (a) Western blot analysis showed BRCA1^{5382insC} migrated at ~220 kDa in untreated, (–) H_2O_2, HCC1937 cells. BRCA1^{5382insC} migrated at ~270 kDa in H_2O_2-treated cells, (+) H_2O_2. **(b)** K48-linked ubiquitin moieties (K48-Ubq) were detected at ~220 kDa in untreated cells and correspondingly at ~270 kDa in H_2O_2-treated cells. **(c)** BARD1 (~87 kDa) was detected in untreated HCC1937 cells in the same fractions as BRCA1^{5383insC}, and was detected in H_2O_2-treated cells at ~87 kDa. The elution profiles are more complex under oxidative conditions. Nuclear extract (NE); Eluted material (E).

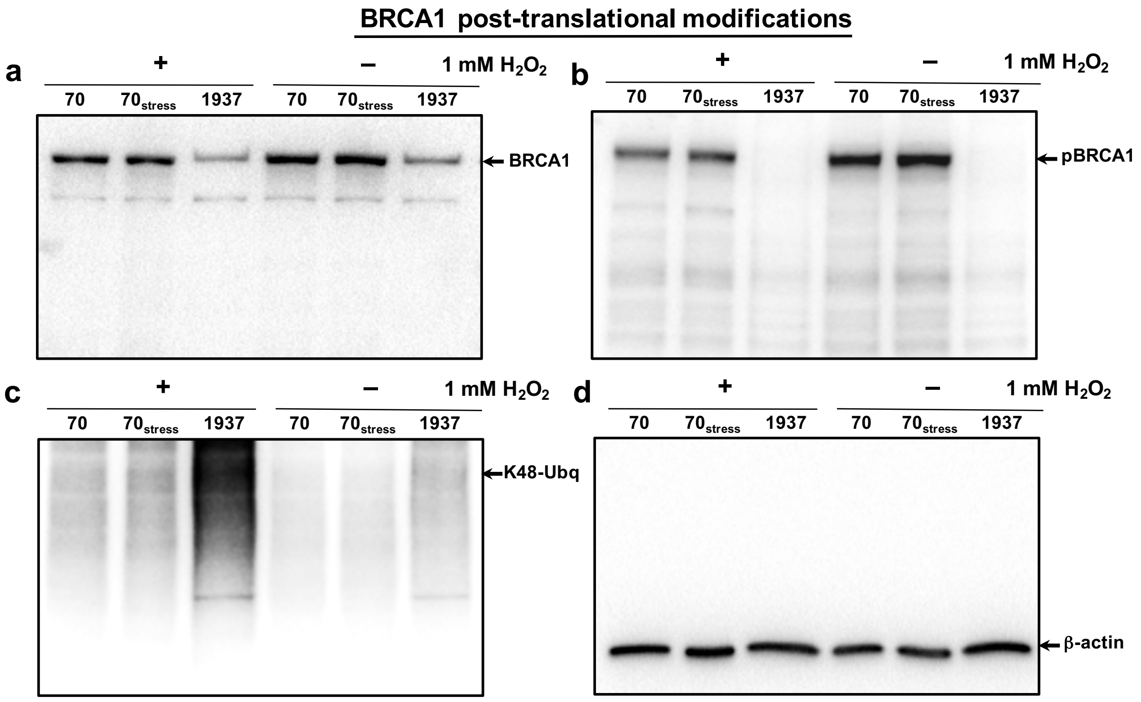

Supplementary Figure 4. Post-translational modifications detected in the nuclear material of various breast cancer cells under oxidative conditions. The nuclear material of H_2O_2-treated and untreated cells including HCC70, $HCC70_{stress}$, and HCC1937 lines was assessed by western blot analysis. **(a)** BRCA1 was present in each fraction. **(b)** Phosphorylated BRCA1 (pBRCA1) was detected in HCC70 and $HCC70_{stress}$ cells with and without H_2O_2 treatment using antibodies against phosphorylated residue S1524. pBRCA1 was not detected in HCC1937 cells. **(c)** K48-linked ubiquitin moieties were elevated in all cells upon H_2O_2 treatment especially the HCC1937 cells. **(d)** β-actin served as a loading control.
